# Combined chitosan and Dan-shen injection for long-term tubal patency in fallopian tube recanalization for infertility

**DOI:** 10.1007/s13346-018-00611-0

**Published:** 2019-01-04

**Authors:** Chen Huang, Xueping He, Wenfeng Luo, Hanwei Chen, Yi Huang

**Affiliations:** 1 Jinan University, 601 Huangpu Avenue West, Tianhe District, Guangzhou, 511400 China; 2 Department of Radiology, Guangzhou Panyu Central Hospital, 8 East Fuyu Road Qiaonan Street, Panyu District, Guangzhou, 511400 China; 3 Medical Imaging Institute of Panyu, 8 East Fuyu Road Qiaonan Street, Panyu District, Guangzhou, 511400 China

**Keywords:** Chitosan injection, Dan-shen injection, Fallopian tube obstruction, Fallopian tube recanalization, Adhesion prevention and control

## Abstract

To prospectively study the efficacy of different anti-adhesion agents for the prevention of tubal obstruction after recanalization, this trial was approved by our hospital ethics committee. Four hundred patients with fallopian tube obstruction were randomly assigned to four groups. The control group underwent recanalization alone, whereas the other groups were injected with chitosan, Dan-shen, or combined chitosan and Dan-shen after recanalization. The tubal patency rate in all four groups was recorded after 12 day, 3 months, and 12 months. The pregnancy rates were noted after 12 months. The recanalization rates after 1 day in the control, chitosan, Dan-shen, and combined chitosan and Dan-shen groups were 94.1, 97.1, 96.5, and 98.2%, respectively (*p* = 0.18, *p* > 0.05). The rates of tubal patency after 3 months were significantly higher in the combined chitosan and Dan-shen (96.5%), chitosan (88%), and Dan-shen (85.2%) groups compared with the control group (73.9%) (*p* = 0.0001, *p* < 0.05). The recanalization rate and intrauterine pregnancy rate after 12 months was significantly higher in the combined chitosan and Dan-shen group (93.8 and 63.9%, respectively) compared with the other groups (control 39 and 30.6%, chitosan 78.4 and 46.9%, and Dan-shen 77.3 and 43.3%) (*p* = 0.0029 and *p* = 0.0001, *p* < 0.05). Chitosan, Dan-shen, or a combination of the two compounds could be effective for preventing tubal obstruction after interventional recanalization, possibly increasing the rate of pregnancy in affected women. The combined chitosan and Dan-shen injection has unique advantages in the interventional recanalization of obstructed fallopian tubes.

## Introduction

The global rate of infertility in women is approximately 15.5% and the incidence is increasing [1]. Fallopian tube obstruction can lead to reduced fertility among affected women, accounting for 25 to ~ 35% of all causes of infertility [2]. These causes include chronic salpingitis and sexually transmitted diseases (STDs), and adhesions caused by uterine and abdominal cavity surgery. Although microsurgery is becoming more common (leading to a reduction in adhesions caused by intraabdominal cavity laparotomy), adhesions cannot be completely prevented through the microsurgical technique [3].

Selective salpingography is conducted using a coaxial catheter directed over a guide wire, followed by interventional recanalization for tubal obstruction. However, postoperative re-occlusion of the fallopian tube lumen can readily occur due to its complicated pathology, resulting in a low rate of intrauterine pregnancy following the treatment [4].

Chitosan is a compound produced by the deacetylation of chitin, a compound found in the exoskeletons of crustaceans, such as shrimp and crabs, and in insects. The chemical name of chitosan is beta-(1–4)-2-amino-2-deoxy-D-glucose. Chitosan exists in gray white or light yellow translucent amorphous, and it is odorless and tasteless. It is also insoluble in water. The basic unit of chitosan is glucosamine. It has good biocompatibility, biodegradability, and biological activity.

Research has shown that the chitosan injection possesses bacteriostatic features, as well as anti-adhesion and hemostasis properties, suggesting a potential to decrease tubal postoperative re-occlusion in infertile women and subsequently increase the pregnancy rate.

Dan-shen, comprised of the dried roots and rhizomes of *Salvia miltiorrhiza* Bge, is a type of traditional Chinese medicine. The chemical constituents of Dan-shen are either fat-soluble or water-soluble. The fat-soluble constituents include tanshinone, tanshinone II, tanshinone IIB, cryptotanshinone, hydroxytanshinone, and methyltanshinonenate, and the water-soluble constituents include salvianolic acid A, salvianolic acid B, and salvianic acid A.

Research has shown that *Salvia miltiorrhiza* has many pharmacological activities, e.g., anti-inflammatory, anti-peptic ulcer, improving peripheral circulation, and anti-hepatic fibrosis, as well, and improves tubal function and reduces the probability of postoperative re-occlusion and tubal pregnancy, ultimately improving the potential for an intrauterine pregnancy [5].

Between 2013 and 2016, we attempted to find more effective methods for preventing tubal postoperative re-occlusion and increasing the success rate of intrauterine pregnancy. In this work, we reached the conclusion that the combination of chitosan and Dan-shen is stable and produces a synergistic effect. We investigated the application of the chitosan injection, Dan-shen injection, and combined chitosan and Dan-shen injection for fallopian tube recanalization, in addition to the mechanism used by the combined chitosan and Dan-shen injections to prevent and cure postoperative tubal re-occlusion and promote the synergistic effect of tissue repair.

## Materials and methods

This prospective study was conducted at our hospital between 2013 and 2016. The study was approved by the medical ethics committee of Guangzhou Panyu Central Hospital (Reference number: P20120038). Informed consent was obtained from all patients prior to participation in the study.

### Patient selection

The Inclusion criteria were as follows: (1) females of child-bearing age (between 22 and 35 years); and (2) a diagnosis of Fallopian tube isthmus or interstitial obstruction without adhesions of the fimbriated extremity of the tube, determined by laparoscopic examination or salpingography.

The exclusion criteria included (1) diagnosis of acute genital/pelvic infection, congenital physiological defects or malformation, infertility caused by genetic factors, endocrine factors (follicle stimulating hormone (FSH) or luteinizing hormone (LH) or other hormones) or immune factors; (2) diagnosis of endometriosis, myoma of the uterus, hypoplasia of the uterus, or genital or pelvic tuberculosis; (3) patient with a male partner with diagnosed dysfunction of the reproductive system; (4) allergy to the study drugs; or (5) incomplete information or inability to take medication according to study instructions.

A total of 400 eligible patients were enrolled between 2013 and 2016. Through salpingography, 328 fallopian tubes with obstruction at the isthmus and 364 tubes with obstruction at the interstitial portion were discovered. The patients were randomized into four groups of 100 patients each, including a control group, chitosan group, Dan-shen group, and a combined chitosan and Dan-shen injection group. The groups were blinded to the nature of the anti-adhesion agents they each received. The control group underwent coaxial catheter interventional recanalization alone, whereas the other groups underwent the recanalization followed by injection with chitosan, Dan-shen, and combined chitosan and Dan-shen.

### Procedure

#### Materials and equipment

The principal equipment and materials included the OEC 9600 C-arm X-ray machine (General Electric), fallopian tube recanalization double-balloon catheter, 5.5F type J catheter, 3.0 F MICRO, 0.18 Micro guidewire, and 0.035 guide wire (Cook, Inc., Bloomington, IN, USA). The primary medications used included injectable chitosan (Shanghai Qisheng Bas Co., Ltd., product lot number: 12080311) and injectable Dan-shen (Shanghai Qisheng Bas Co., Ltd., product lot number: 12080563).

#### Procedure

Interventional recanalization therapy was performed within 3–7 days after the end of menstruation as follows: Under the digital subtraction angiography (DSA), the front of the balloon was inserted through the cervix and into the uterine cavity. After dilatation of the balloon, a 20-mL dose of contrast agent (Iopromide, Ultravist, Bayer, Germany) was injected into the uterine cavity to demonstrate its shape and the position of the cornu uteri, as well as to confirm the obstructed portion(s) of the fallopian tube. The guidewire and catheter were subsequently directed to the affected side of the cornu uteri, with the tip of the catheter entering the deep cornu uteri over the guide wire. After insertion of a microcatheter, the coaxial catheter system was finally established to clear the fallopian tube obstruction. A 20-mL dose of contrast agent was subsequently injected once more to confirm tubal patency. To complete the therapy, each patient received a liquid mixture comprising 80,000 U gentamicin, 1000 U chymotrypsin, 10 ml 2% lidocaine, and 10 mg dexamethasone injected into both fallopian tubes through the catheter. Finally, 2 mL of chitosan was used for the chitosan injection group, whereas 10 mL of Dan-shen was used for the Dan-shen injection group. The combined chitosan and Dan-shen injection group comprised a mixture of chitosan and Dan-shen solution at a 1:5 ratio (2 mL chitosan mixed with 10 mL Dan-shen).

Lastly, the levels of specific reproductive hormones (follicle stimulating hormone, luteinizing hormone, estrogen, and progesterone) were measured and assessed.

### Follow-up

All the patients were followed to assess maintenance of tubal patency, using hydrotubation outcome measures after 3 months and salpingography after 12 months. Ultrasound was used to ascertain pregnancy, which was determined to be intrauterine rather than ectopic in the postoperative 12 months. Those patients who did not return for follow-up examinations were contacted by telephone.

### Definitions

#### Observation targets

##### Preoperative observation targets

The results of a sex hormone examination, leukorrhea examination, type B ultrasound examination of the pelvic cavity, laparoscopic examination, hysteroscopic examination, and hysterosalpingography examination.

##### Intraoperative observation targets

The cavum uteri shape, location of the Fallopian tube obstruction, and the recanalization rate of the interventional therapy for Fallopian tube obstruction.

##### Postoperative observation targets

The tubal patency rate in the 3 months postprocedure, the tubal patency rate (in nonpregnant patients**)** in the subsequent 12 months, and the pregnancy rate (except for in vitro fertilization) in the subsequent 12 months.

#### Outcome measures [5]

##### Salpingography

i. Unobstructed: Both sides of the fallopian tubes can be shown clearly during the procedure and visible contrast agent can be seen diffusing through the pelvic cavity.
ii. Completely obstructed: There is partial or no development of the middle and distal end of the fallopian tube, and no contrast agent can be seen diffusing through the pelvic cavity.
iii. Partially obstructed: The contrast agent can be seen discharging from the fallopian tube and partially flowing into the pelvic cavity in an inhomogeneous manner. In addition, the fallopian tubes can be seen forming irregular configurations.

##### Hydrotubation (tubal flushing) outcome measures

i. Unobstructed: In the hydrotubation treatment conducted 3 months after the procedure, a total of 20 mL normal saline can be injected without resistance being felt, with the pressure being maintained under 60–80 mmHg (1 mmHg = 0.133 kPa). Alternatively, the resistance is present at the beginning of the injection before disappearing, without liquid flowing back and patients experiencing malaise.
ii. Completely obstructed: Resistance can be felt after barely injecting 5 mL of normal saline, with the pressure rising, as shown on a pressure gauge. In addition, the patient can feel the pain of distension in the lower abdomen and the liquid flows back to the injector after the injection is stopped.
iii. Partially obstructed: Resistance is present during the injection, but the liquid can still be injected by applying increased pressure, signifying that mild adhesions are being separated. In addition, the patients can feel the pain of abdominal distension.

### Statistical analysis

The data from each group were collected and analyzed using SPSS 11.5 software (IBM SPSS China, Shanghai, china). The enumeration data were checked and corrected using the chi-squared test (*x*^2^), whereas the measurement data were calculated as mean (*X*) ± standard deviation (s). In addition, the one-tailed *t* test was used, with the differences showing significance only if the *p* value was < 0.05.

## Results

There were no statistically significant differences in the mean ages of the study groups (control, chitosan injection, Dan-shen injection, and combined chitosan and Dan-shen injection) (*p* > 0.05). The differences within each hormone group (estrogen and progesterone, and FSH and LH) before and after surgery also showed no statistical significance (*p* > 0.05). In addition, the differences among the groups in the preoperative vaginal fluid, preoperative uterine conditions, the location of the obstruction, and the preoperative positions of one or both sides of the fallopian tube obstruction also showed no statistically significant difference (*p* > 0.05). An analysis of the ages and hormones of each group is shown in Table 1, 2, 3, 4, 5, and 6**)**.

**Table 1:** Analysis of patient ages

	Control group	Chitosan injection group	Dan-shen injection group	Combined chitosan and Dan-shen injection group	*p* value	Method
*N*	100	100	100	100		
Mean ± SD	30.05 ± 5.18	30.08 ± 4.64	29.97 ± 4.37	30.03 ± 5.32	0.991	ANOVA
95% CI (L~U)	29.54~30.56	29.62~30.53	29.54~30.40	29.29~30.77		
Min~max	20.00~46.00	19.00~42.00	22.00~46.00	20.00~48.00		
Median	30.00	30.00	29.00	29.00

**Table 2:** Analysis of preoperative and postoperative estrogen

	Control group	Chitosan injection group	Dan-shen injection group	Combined chitosan and Dan-shen injection group	*p* value	Method
Preoperative estrogen (ng/l)						
Mean ± SD	178.49 ± 39.59	178.51 ± 37.77	178.49 ± 29.90	178.50 ± 34.76	1.000	ANOVA
95%CI (L~U)	174.59~182.38	174.80~182.22	175.55~181.43	173.65~183.34		
Min~max	86.00~301.00	89.00~301.00	110.00~258.00	110.00~265.00		
Median	185.50	187.00	185.00	178.00		
Postoperative estrogen (ng/l)						
Mean ± SD	178.51 ± 40.03	178.54 ± 36.55	178.52 ± 30.89	178.49 ± 38.84	1.000	ANOVA
95%CI (L~U)	174.57~182.44	174.95~182.13	175.48~181.55	173.07~183.91		
Min~max	83.00~302.00	91.00~303.00	103.00~257.00	20.00~301.00		
Median	184.00	187.00	186.00	177.50

**Table 3:** Analysis of preoperative and postoperative progesterone

	Control group	Chitosan injection group	Dan-shen injection group	Combined chitosan and Dan-shen injection group	*p* value	Method
Preoperative progesterone (μg/l)						
Mean ± SD	14.43 ± 6.42	14.41 ± 6.43	14.42 ± 6.06	14.44 ± 5.26	1.000	ANOVA
95%CI (L~U)	13.80~15.06	13.77~15.04	13.83~15.02	13.70~15.17		
Min~max	1.60~25.30	1.60~24.50	2.80~24.50	2.50~24.50		
Median	14.30	14.30	14.00	14.20		
Postoperative progesterone (μg/l)						
Mean ± SD	14.43 ± 6.43	14.40 ± 6.50	14.43 ± 6.05	14.46 ± 5.47	1.000	ANOVA
95%CI (L~U)	13.80~15.06	13.76~15.04	13.83~15.02	13.70~15.23		
Min~max	1.50~25.10	1.80~24.70	1.70~24.70	1.70~24.70		
Median	14.60	14.60	14.05	14.00

**Table 4:** Analysis of preoperative and postoperative FSH

	Control group	Chitosan injection group	Dan-shen injection group	Combined shitosan and Dan-shen injection group	*p* value	Method
Preoperative FSH (U/l)						
Mean ± SD	9.00 ± 3.56	9.01 ± 3.87	9.02 ± 3.81	9.02 ± 3.04	1.000	ANOVA
95% CI (L~U)	8.65~9.35	8.63~9.39	8.64~9.39	8.60~9.44		
Min~max	3.60~19.70	3.30~19.40	3.30~19.20	3.30~19.20		
Median	8.40	8.20	8.20	9.20		
Postoperative FSH (U/l)						
Mean ± SD	9.01 ± 3.75	9.03 ± 3.63	9.00 ± 3.86	9.03 ± 3.43	0.999	ANOVA
95% CI (L~U)	8.64~9.38	8.67~9.39	8.62~9.37	8.55~9.50		
Min~max	3.60~19.90	3.40~19.70	3.10~19.40	3.10~19.30		
Median	8.40	8.40	8.20	9.10

**Table 5:** Analysis of preoperative and postoperative LH

	Control group	Chitosan injection group	Dan-shen injection group	Combined chitosan and Dan-shen injection group	*p* value	Method
Preoperative LH (U/l)						
Mean ± SD	29.06 ± 14.86	29.07 ± 16.58	29.09 ± 16.70	29.04 ± 17.48	1.000	ANOVA
95% CI (L~U)	27.60~30.52	27.44~30.70	27.44~30.73	26.60~31.48		
Min~max	10.50~76.90	10.00~76.90	10.00~76.90	10.50~76.90		
Median	23.40	22.70	22.50	22.00		
Postoperative LH (U/l)						
Mean ± SD	29.04 ± 15.09	29.05 ± 16.19	29.08 ± 16.70	29.03 ± 17.15	1.000	ANOVA
95% CI (L~U)	27.56~30.53	27.46~30.64	27.44~30.72	26.64~31.42		
Min~max	9.90~76.70	10.20~77.10	10.20~76.70	10.30~77.20		
Median	22.90	22.80	22.50	22.10

**Table 6:** Analysis of preoperative positions of the obstruction in one or both of the fallopian tubes

	Control group (%)	Chitosan injection group (%)	Dan-shen injection group (%)	Combined chitosan and Dan-shen injection group (%)
Obstruction in one fallopian tube				
Interstitial portion	13 (56.5)	14 (56.0)	14 (53.8)	13 (50.0)
Ampulla portion	4 (17.3)	3 (12.0)	5 (19.2)	6 (23.1)
Isthmus portion	6 (26.0)	8 (32.0)	7 (26.9)	7 (26.9)
Total	23	25	26	26
S = 0.568; *p* = 0.997; R × C				
Obstruction in both fallopian tubes				
Interstitial portion	39 (53.4)	38 (50.6)	38 (52.1)	40 (53.3)
Ampulla portion	13 (17.8)	14 (18.7)	13 (17.8)	12 (16.0)
Isthmus portion	21 (28.8)	23 (30.7)	22 (30.1)	23 (30.7)
Total	73	75	73	75
S = 1.270; *p* = 0.973; R × C				

Twelve of the enrolled patients were lost to follow-up, including two in the control group, four in the chitosan injection group, three in the Dan-shen injection group, and three in the combined chitosan and Dan-shen injection groups. There were no statistically significant differences between the patients who completed the study and those who were lost to follow-up with respect to age, hormone, preoperative vaginal fluid, preoperative uterine conditions, the location of the obstruction, and the preoperative positions of one or both sides of the fallopian tube obstruction.

### Analysis of the tubal recanalization success rates 1 day after interventional recanalization

**Fig. 1:**
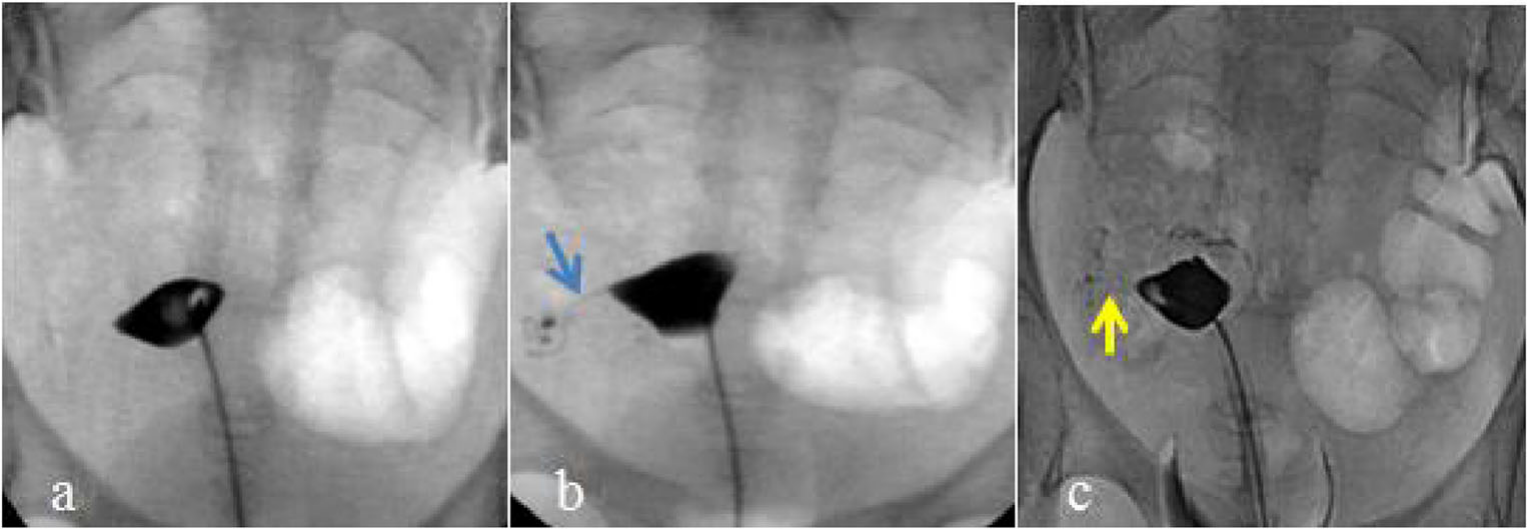
Young female in the control group (26 years of age). **a** Selective salpingography showed the right fallopian tube completely obstructed at the preoperative stage. **b** Selective salpingography showed the right fallopian tube partially obstructed 1 day after interventional recanalization. The blue arrow shows the right tubal partially recanalized. **c** Selective salpingography showed the right fallopian tube partially obstructed in the postoperative 12 months. The yellow arrow shows the right tube partially recanalized

**Fig. 2:**
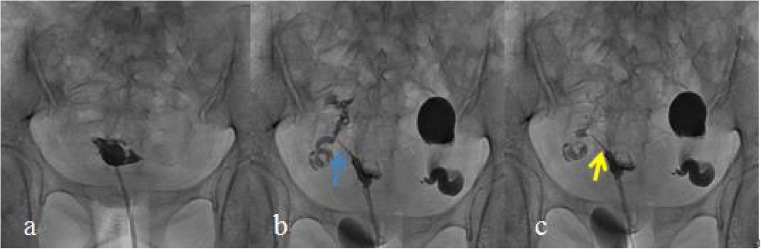
Young female in the chitosan injection group (32 years of age). **a** Selective salpingography showed the right fallopian tube completely obstructed at the preoperative stage. **b** Selective salpingography showed the right fallopian tube unobstructed 1 day after interventional recanalization. The blue arrow shows the right tubal completely recanalized. **c** Selective salpingography showed the right fallopian tube partially obstructed in the postoperative 12 months. The yellow arrow shows the right tube partially recanalized

**Fig. 3:**
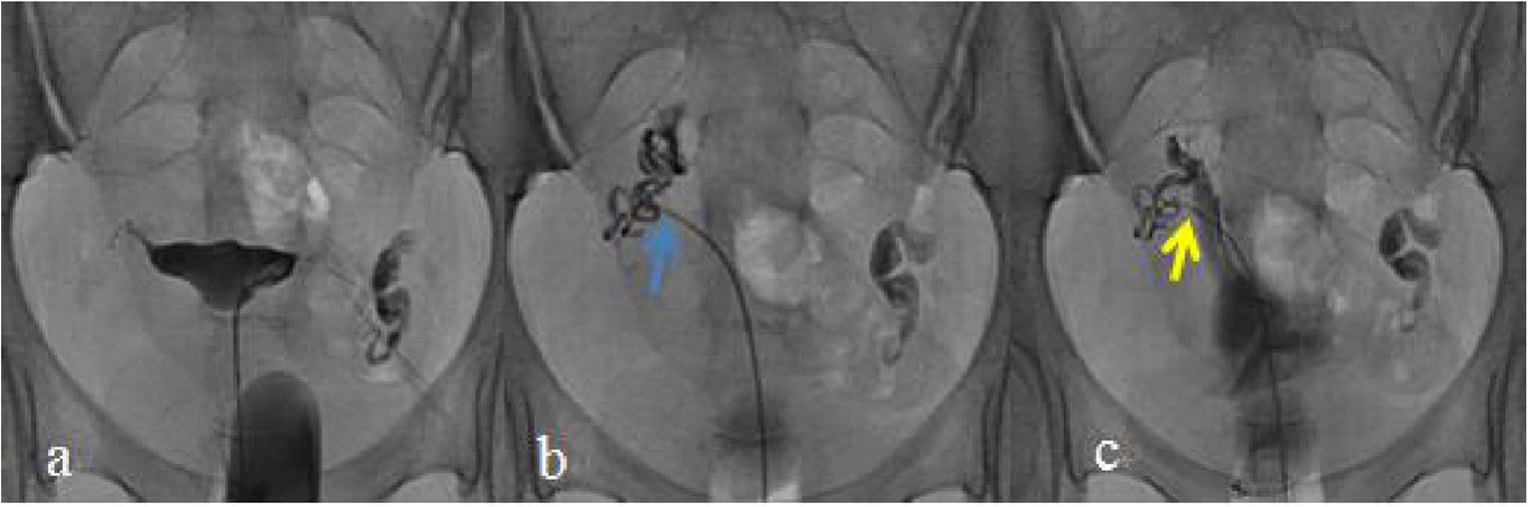
Young female in the Dan-shen injection group (30 years of age). **a** Selective salpingography showed the right fallopian tube partially obstructed at the preoperative stage. **b** Selective salpingography showed the right fallopian tube unobstructed 1 day after interventional recanalization. The blue arrow shows the right tubal completely recanalized. **c** Selective salpingography showed the right fallopian tube unobstructed in the postoperative 12 months. The yellow arrow shows the right tube completely recanalized

**Fig. 4:**
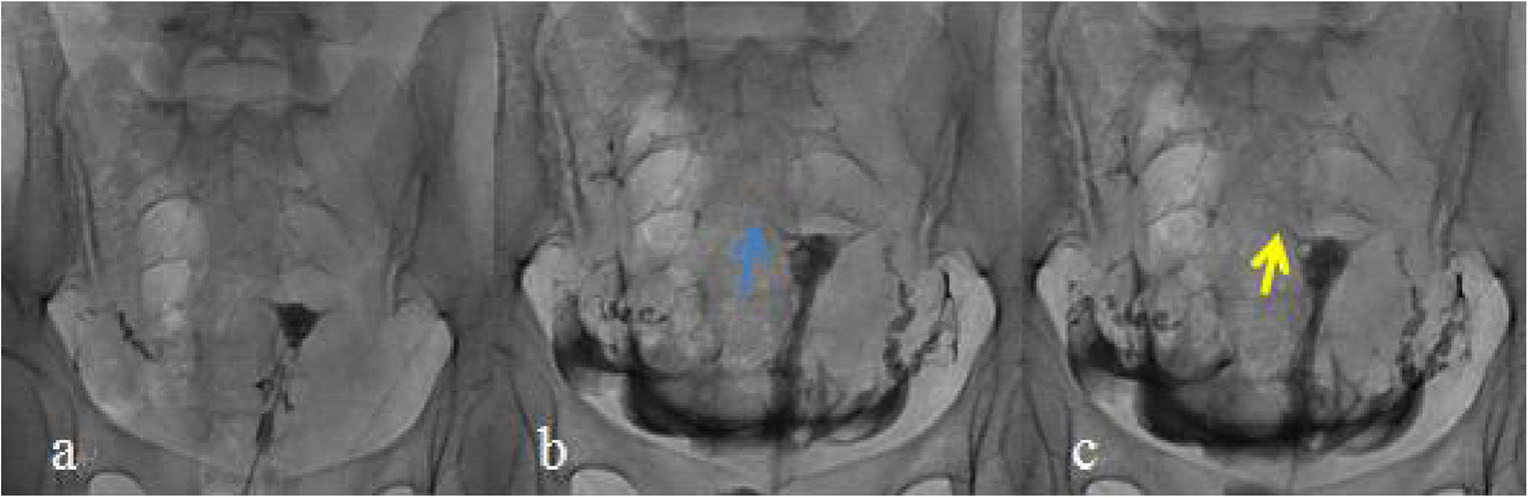
Young female in the combined chitosan and Dan-shen injection group (28 years of age). **a** Selective salpingography showed the left fallopian tube completely obstructed at the preoperative stage. **b** Selective salpingography showed the right fallopian tube unobstructed 1 day after interventional recanalization. **c** Selective salpingography showed the right fallopian tube unobstructed in the postoperative 12 months

A total of 800 fallopian tubes were evaluated in 400 patients, and 692 tubes were found to be obstructed before the procedure. Afterwards, the tubal recanalization rates were highest in the combined chitosan and Dan-shen injection group (above 98%) and lowest in the control group (above 94%), and despite this trend, the differences were not statistically significant (*p* = 0.18, *p* > 0.05, Table 7). Figures 1, 2, 3, and 4 show a representative patient from each of the four treatment groups, respectively, at the preoperative stage, 1 day after interventional recanalization and 12 months postop. The obstructed left fallopian tubes became patent after operation.

**Table 7:** Analysis of fallopian tube recanalization rate 1 day after the treatment

	Preoperative obstructed	Postoperative unobstructed	Postoperative partially obstructed	Postoperative obstructed	Recanalization success rate (%)	95% CI
Control	169	159	6	4	94.1	(90.5, 97.6)
Chitosan injection	175	170	3	2	97.1	(94.7, 99.6)
Dan-shen injection	172	166	3	3	96.5	(93.8, 99.3)
Combined chitosan and Dan-shen injection	176	173	2	1	98.2	(96.4, 100)

Recanalization success rate = number of postoperative unobstructed tubes/number of preoperative obstructed tubes × 100%

### Analysis of the hydrotubation patency rate at 3 months after the treatments

The success rates for the combined chitosan and Dan-shen injection group were highest among the four injection groups (above 96%), and all the rates were significantly higher than the hydrotubation patency rate of the control group (73.9%) (*p* = 0.0001, *p* < 0.05), as shown in Table 8.

**Table 8:** Analysis of the hydrotubation patency rate 3 months after the treatment

	Preoperative obstructed	Postoperative unobstructed	Postoperative partially obstructed	Postoperative obstructed	Patency rate (%)	95% CI
Control	169	125	20	24	73.9	(67.3, 80.6)
Chitosan injection	175	154	12	9	88.0	(83.2, 92.8)
Dan-shen injection	172	145	15	12	85.2	(78.9, 89.7)
Combined chitosan and Dan-shen injection	176	170	4	2	96.5	(92.3, 100)

Patency rate = number of postoperative unobstructed tubes/number of preoperative obstructed tubes × 100%

### Analysis of the tubal patency rate in nonpregnant patients in the postoperative 12 months

The tubal patency rate in the combined chitosan and Dan-shen injection group was substantially higher than the rate in the control group (93.8% vs. 39%), a difference that was statistically significant (*p* = 0.0029, *p* < 0.05), as shown in Table 9. The trend in the tubal patency rate for the other treatment groups was similar, being higher than the rate for the control group.

**Table 9:** Analysis of the tubal patency rate (in non-pregnant patients) in the postoperative 12 months

	Total of non-pregnant patients	Postoperative unobstructed	Postoperative partially obstructed	Postoperative obstructed	Patency rate (%)	95% CI
Control	136	53	22	61	39.0	(31.2, 47.4)
Chitosan injection	102	80	12	10	78.4	(70.5, 86.2)
Dan-shen injection	110	85	14	11	77.3	(69.5, 84.8)
Combined chitosan and Dan-shen injection	70	66	2	2	93.8	(86.4, 100)

Patency rate = the number of the postoperative unobstructed tubes/the number of follow-up patients the tubes × 100%

### Analysis of the pregnancy rate in the postoperative 12 months

The pregnancy rates in the postoperative 12 months in the combined chitosan and Dan-shen injection group was 63.9%, which was higher than the pregnancy rate in the control group (30.6%), and the other treatment groups, which demonstrated rates lower than the combined treatment compound. The difference was statistically significant (*p* = 0.0001, *p* < 0.05), as shown in Table 10.

**Table 10:** Analysis of the pregnancy rate in the postoperative 12 months

	Pregnant	Not pregnant	Pregnancy rate (%)	95% CI
Control	30	68	30.6	(13.1, 48.2)
Chitosan injection	45	51	46.9	(27.7, 66.1)
Dan-shen injection	42	55	43.3	(24.1, 62.5)
Combined chitosan and Dan-shen injection	62	35	63.9	(44.8, 83.1)

Pregnancy rate = number of pregnant patients/follow-up number of patients × 100%

## Discussion

Interventional recanalization has now become one of the most important treatment methods for salpinx obstructive infertility due to the procedure’s positive safety profile, its efficacy, and the fact that interventional recanalization is minimally invasive. This technique not only allows an evaluation of the architecture and diseases of the uterus and fallopian tubes but also facilitates the clearing of any proximal tubal obstruction [6].

However, tubal postoperative re-occlusion or endometrial function loss can easily give rise to tubal pregnancies and result in a low likelihood of conception. Cellular debris is thought to be the most common cause of fallopian tube occlusion. The recanalization rate of the traditional tubal interventional procedure is approximately 90%, with a high postoperative re-occlusion rate of 20–50% [7]. The pregnancy rate following fallopian tube recanalization is about 30% [8], and the rate of intrauterine pregnancy is about 28.9%% [9]. Seyam [10] showed a cumulative pregnancy rate is about 26% following any of two different FTR procedures. Al-Omari MH [11] found that younger patients with secondary infertility lasting for less than 5 years have an extremely high chance of becoming pregnant following FTR.

Thus, prevention of fallopian tube re-occlusion has become the principal focus of the clinical therapy for tubal obstructive infertility to increase the pregnancy rate in affected patients.

Our previous study found that the tubal postoperative recanalization rate was higher in patients who underwent catheter interventional recanalization alone compared with patients with chitosan injection treatment, with the re-occlusion rate being only 4.26% 3 months after the treatment [12]. The present study found that the combined chitosan and Dan-shen injection had a positive clinical effect on the prevention of tubal postoperative re-occlusion and on increasing the pregnancy rate. This is because the chitosan part of the injection leads to bacteriostasis, anti-adhesion, and hemostasis, effectively decreasing tubal postoperative re-occlusion. Choi’s [13] experiments on mice showed that chitosan could improve the function of the ovary and fallopian tubes. Chung [14] demonstrated that postoperative adhesions can be prevented by chitosan. Liu Xin Lei [15] reported a re-occlusion rate of 8.9% when the chitosan injection was applied, preventing an inflammatory reaction, in addition to its other effects. Huang Yi [16] reported that the recanalization rate, tubal patency rate in the postoperative 3 months, and the pregnancy rate in the postoperative 12 months in the chitosan injection group were 95.7%, 91.7%, and 48.1%, respectively. Zhao Bo [17] showed that the patency rate in the postoperative 12 months of the chitosan group was 91.3%, whereas the pregnancy rate was 72.5% over the same period. Thus, the mechanism by which chitosan prevents postoperative tissue adhesions may include the following:

1. Chitosan has the biological characteristics of selectively promoting the growth of epithelial and endothelial cells and inhibiting the growth of fibroblasts, thus promoting the physiological repair of tissues, inhibiting scar formation, and reducing tissue adhesions.
2. Chitosan has local hemostatic effect and inhibits the formation of fibrin bundles, thus reducing the tissue adhesions caused by hematomas.
3. Chitosan functions as a lubrication and biological barrier, effectively preventing adhesions.

The Dan-shen injection had a positive clinical effect on the maintenance of tubal patency when applied with the interventional recanalization. As shown in the literature [18], the Dan-shen injection is not only an adrenergic α-receptor agonist but also promotes blood circulation with the goals of reducing blood stasis, relieving pain, promoting the recovering of the uterine tubal epithelium and cilia [19], increasing blood flow to improve ischemia and hypoxia in inflamed fallopian tubes, and inhibiting the excessive damage of reactive oxygen radicals to protect epithelial cells in fallopian tubes. The Dan-shen injection could also reduce the abnormal expression of smad3 mRNA and caused anti-tissue adhesion and fibrosis [20].The Dan-shen injection also promotes lysis of adhesions by anti-fibrosis and inhibits the synthesis of collagen, in addition to inhibiting the growth of bacteria such as *Staphylococcus aureus*, Mycobacterium tuberculosis H37Rv, *Escherichia coli*, Bacillus proteus, and subserotypes of Shigella flexneri [21].Other research studies have shown that the tubal patency rate in the postoperative 12 months was 93.7%, whereas the intrauterine pregnancy rate was 46.0% [18]. Hu Wenjun [22] showed that the re-occlusion rate and pregnancy rate of the Dan-shen injection combined with the 0_3_ perfusion group were 12.9 and 46.8%, respectively.

We believe that the application of the combined chitosan and Dan-shen injection (rather than administering them separately) has better efficacy for the prevention of tubal re-occlusion and improves the probability of a pregnancy occurring. We studied the stability of the combined chitosan and Dan-shen injection using high performance liquid chromatography to determine the chitosan content changes before and after mixing with the Dan-shen injection. By carefully studying the chromatogram components, such as the primary component relative to the peak area measurement, precision results of their physical nature and statistical analysis, we reached the conclusion that the combination is stable and produces a synergistic effect. In addition, the combination produces few complications and high operability, all properties that have not been reported in the literature previously.

The reason for this phenomenon is that the complex dimensional structure of chitosan, as a polymeric compound, can serve as a carrier for the active ingredients in the Dan-shen injection. Furthermore, chitosan’s capacity to remain at a single local position in the body for more than 3 weeks, allows the active ingredients of the Dan-shen injection to be released more slowly. Based on the results of this study, the application of the combined chitosan and Dan-shen injection in interventional recanalization substantially improves the recanalization rate (98.2%, vs. 97.1% in the chitosan injection group and 96.5% in the Dan-shen injection group).

The tubal patency rate in the postoperative 12 months in the combined chitosan and Dan-shen injection group (93.8%) was significantly higher than the rates seen in the control group (39%), chitosan injection group (78.4%), and the Dan-shen injection group (77.3%). The pregnancy rate in the postoperative 12 months of the combined chitosan and Dan-shen injection group (63.9%) was also significantly higher than that of the control group (30%), chitosan injection group (52%), and the Dan-shen injection group (49%). These results indicate that the combined chitosan and Dan-shen injection has a positive effect on the probability of tubal patency and pregnancy within the postoperative 12-month period. The related theory and technology could be promoted at basic-level hospitals.

One limitation of our study is that all the groups that received anti-inflammatory drugs. It is difficult to determine whether the administered anti-inflammatory drugs affected the patency of the fallopian tubes. Another limitation of our study is that the pharmacology of the combined chitosan and Dan-shen injection was not clear. We plan to investigate the mechanism of their interaction in a future study.

In conclusion, chitosan, Dan-shen, and a combination of the two compounds can effectively reduce the rate of tubal postoperative re-occlusion and subsequently increase the pregnancy rate. The combined chitosan and Dan-shen injection may have unique advantages in the interventional recanalization of obstructed fallopian tubes.
